# Molecular Weight-Dependent Immunostimulative Activity of Low Molecular Weight Chitosan via Regulating NF-κB and AP-1 Signaling Pathways in RAW264.7 Macrophages

**DOI:** 10.3390/md14090169

**Published:** 2016-09-20

**Authors:** Bin Zheng, Zheng-Shun Wen, Yun-Juan Huang, Mei-Sheng Xia, Xing-Wei Xiang, You-Le Qu

**Affiliations:** 1 Zhejiang Provincial Key Engineering Technology Research Center of Marine Biomedical Products, School of Food Science and Pharmaceutics, Zhejiang Ocean University, Zhoushan 316022, China; 6369958@163.com; 2 Zhejiang Marine Development Research Institute, Zhoushan 316022, China; 3 Zhoushan Hospital (Hospital of Chinese Medicine and Orthopedics), Zhoushan 316000, China; zsdrlz@126.com; 4 Ocean College, Zhejiang University, Zhoushan 316021, China; msxia@zju.edu.cn

**Keywords:** immunostimulative activity, NF-κB/AP-1, molecular weight, low molecular weight chitosans, macrophages

## Abstract

Chitosan and its derivatives such as low molecular weight chitosans (LMWCs) have been found to possess many important biological properties, such as antioxidant and antitumor effects. In our previous study, LMWCs were found to elicit a strong immunomodulatory response in macrophages dependent on molecular weight. Herein we further investigated the molecular weight-dependent immunostimulative activity of LMWCs and elucidated its mechanism of action on RAW264.7 macrophages. LMWCs (3 kDa and 50 kDa of molecular weight) could significantly enhance the mRNA expression levels of COX-2, IL-10 and MCP-1 in a molecular weight and concentration-dependent manner. The results suggested that LMWCs elicited a significant immunomodulatory response, which was dependent on the dose and the molecular weight. Regarding the possible molecular mechanism of action, LMWCs promoted the expression of the genes of key molecules in NF-κB and AP-1 pathways, including IKKβ, TRAF6 and JNK1, and induced the phosphorylation of protein IKBα in RAW264.7 macrophage. Moreover, LMWCs increased nuclear translocation of p65 and activation of activator protein-1 (AP-1, C-Jun and C-Fos) in a molecular weight-dependent manner. Taken together, our findings suggested that LMWCs exert immunostimulative activity via activation of NF-κB and AP-1 pathways in RAW264.7 macrophages in a molecular weight-dependent manner and that 3 kDa LMWC shows great potential as a novel agent for the treatment of immune suppression diseases and in future vaccines.

## 1. Introduction

Chitosan is an abundant, natural linear polysaccharide derived from the deacetylation of chitin from crustaceans, insects and fungi. Chitosan is non-toxic (LD_50_ > 16 g/kg), and non-immunogenic, biodegradable and can be manufactured reproducibly on the basis of GMP guidelines [1]. Chitosan and its derivatives are widely used as biomedical material with an established safety profile in humans such as an experiment mucosal adjuvant [2,3,4] and vaccine adjuvant in mice [5]. Recently, chitosan and its derivatives have attracted more and more attention for its commercial applications in the biomedical, food, and chemical industries. The biomedical applications of various forms of chitosan have long been studied. Chitosan derived from chitin is of high molecular weight, has poor solubility and, ultimately, its therapeutic potential. To address these poor physicochemical properties, more active forms, like trimethylated chitosan and low molecular weight chitosans (LMWCs) have been generated. Chitosan and LMWCs interact readily with various cell receptors due to the presence of amine, acetylated amine and hydroxyl groups, and therefore they could trigger a cascade of interconnected reactions in living organisms resulting in anti-diabetic [6], anti-HIV-1 [7], anti-inflammatory [8], anti-oxidant [9], anti-microbial [10], neuroprotective [11] and anti-angiogenic [12] effects. Chitosan and its derivatives have previously been reported to possess immunological enhancement as a novel adjuvant for vaccine. Chitosan has complex and size-dependent effects on innate and adaptive immune responses including mobilization and activation of innate immune cells and production of cytokines and chemokines [13,14]. Suzuki et al. (1986) reported that different molecular weights of chitosan enhanced immune regulation with the increased water-solubility of chitosan in vivo [15].

Chitosan and its derivatives such as low molecular weight chitosans (LMWCs) have been reported to exert many biological activities, such as antioxidant and antitumor effects. Previous studies reported that LMWCs have dual activities, both immunostimulatory activity in non-induced RAW264.7 [16] and anti-inflammatory activity in induced RAW264.7 cells [17]. However, complex and molecular weight-dependent effects of chitosan remain controversial and the mechanisms that mediate these complex effects are still poorly defined. In our previous study, we found that LMWCs (3 kDa and 50 kDa) elicited a immunomodulatory response in macrophages via regulating the secretion and expression of cytokines in macrophage in a molecular weight- and concentration-dependent manner [18]. However, there was no clear information describing the relationship between molecule weight properties and the mechanism of action of LMWCs on RAW264.7 macrophages. Therefore, we hypothesized that LMWCs may have the potential to augment the immunostimulative activity via NF-κB and AP-1 signaling pathways in molecular-weight-dependent manner. Herein, the present study was carried out to investigate the immunostimulative activity and the mechanism of action of LMWCs on RAW264.7 macrophages by determining the effect on the expression of cytokines and activation of NF-κB and AP-1 signaling pathways.

## 2. Results

### 2.1. Effects of LMWCs on the mRNA Expression Levels of COX-2, IL-10 and MCP-1 in RAW264.7 Macrophages

**Figure 1:**
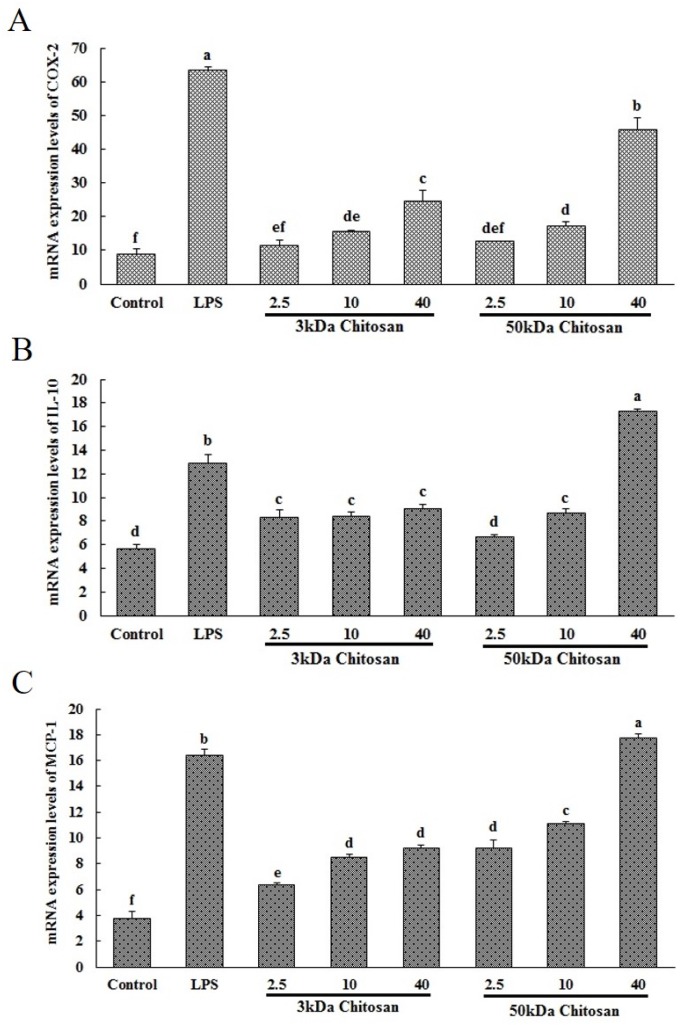
Effect of LMWCs on the mRNA expression levels of COX-2 (**A**), IL-10 (**B**) and MCP-1 (**C**) in RAW264.7 macrophage. Each cell population (1 × 10^6^ cells/mL) was treated with LMWCs (3 kDa and 50 kDa) at the indicated concentrations of 2.5, 10 and 40 μg/mL or LPS (1 μg/mL) for 24 h, respectively. The untreated cells are used as the control. These represent mean values of three independent experiments. Values are presented as means ± SD (*n* = 3, three independent experiments). Bars with different letters (a, b, c, d, e, f) are statistically different (*p* < 0.05).

Inflammatory factors and their signaling molecules play a prominent role in the maturation and function of macrophages. Herein we evaluated the potential of LMWCs to regulate the expression of these mediators in RAW264.7 cells. As shown in Figure 1A–C, the mRNA expression levels of COX-2, IL-10 and MCP-1 in RAW264.7 cells were analyzed by real-time fluorescent quantitative reverse transcription-polymerase chain reaction (RT-PCR). Following LMWC stimulation, the mRNA expression levels of COX-2 and MCP-1 significantly increased in a molecular weight and concentration-dependent manner and 3 kDa chitosan significantly increased the mRNA expression level of IL-10 comparing with untreated cells (*p* < 0.05), which are consistent with the secretion levels of cytokines in the previous study.

### 2.2. Effects of LMWCs on the mRNA Expression Levels of IKKβ in RAW264.7 Macrophages

**Figure 2:**
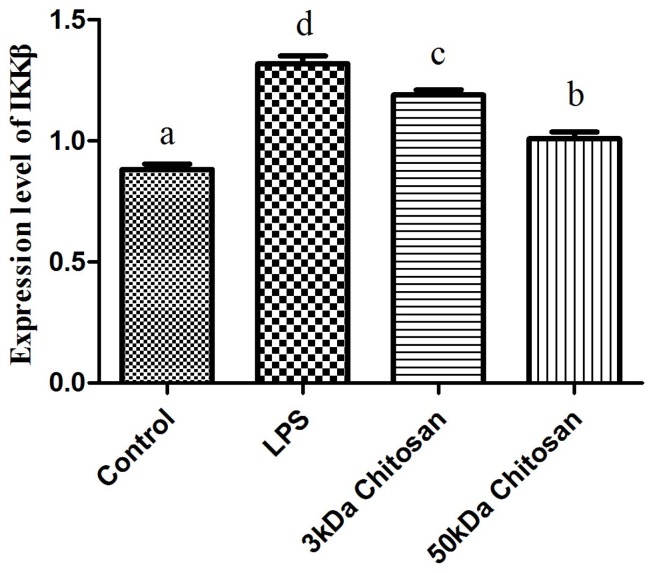
Effects of LMWCs on the mRNA expression levels of IKKβ in RAW264.7 macrophage. Each cell population (4 × 10^5^ cells/mL) was treated with LMWCs (3 kDa and 50 kDa) at the indicated concentrations of 40 μg/mL or LPS (1 μg/mL) for 12 h, respectively. The untreated cells are used as the control. These represent mean values of three independent experiments. Values are presented as means ± SD (*n* = 3). Bars with different letters (a, b, c, d) are statistically different (*p* < 0.05).

The effect of LMWCs on the mRNA expression levels of IKKβ in RAW264.7 macrophages was examined by real-time quantitative RT-PCR. As shown in Figure 2, LMWCs (3 kDa, 50 kDa) significantly enhanced the mRNA expression levels of IKKβ of RAW264.7 cells at the dose (40 μg/mL) compared with the control (*p* < 0.05). Meanwhile, we also found that 3 kDa chitosan significantly promoted the mRNA expression levels of IKKβ compared with that at same dose of 50 kDa chitosan, suggesting that LMWCs significantly induced the mRNA expression levels of IKKβ of macrophages in a molecular weight-dependent manner.

### 2.3. Effect of LMWCs on the mRNA Expression Levels of Key Molecules (TRAF6, JNK1) in RAW264.7 Macrophages

**Figure 3:**
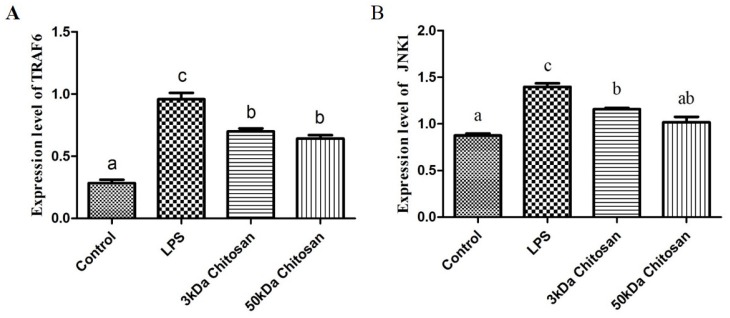
Effects of LMWCs on the mRNA expression levels of key molecules (TRAF6 (**A**), JNK1 (**B**)) from RAW264.7 macrophage. Each cell population (4 × 10^5^ cells/mL) was treated with LMWCs (3 kDa and 50 kDa) at the indicated concentrations of 40 μg/mL or LPS (1 μg/mL) for 12 h, respectively. The untreated cells are used as the control. These represent mean values of three independent experiments. Values are presented as means ± SD (*n* = 3). Bars with different letters (a, b, c) are statistically different (*p* < 0.05).

RAW264.7 cells were treated with LMWCs for 12 h, and the mRNA expression levels of key molecules (TRAF6, JNK1) in RAW264.7 macrophages were detected using real-time quantitative RT-PCR. The addition of LMWCs (3 kDa and 50 kDa) resulted in a remarkable increase in expression levels of TRAF6 and JNK1 compared with untreated cells (*p* < 0.05) (Figure 3), but there were no statistically significant differences between 3 kDa and 50 kDa chitosan (Figure 3A,B).

### 2.4. Effects of LMWCs on the Phosphorylation of IKBα in the RAW264.7 Cells

**Figure 4:**
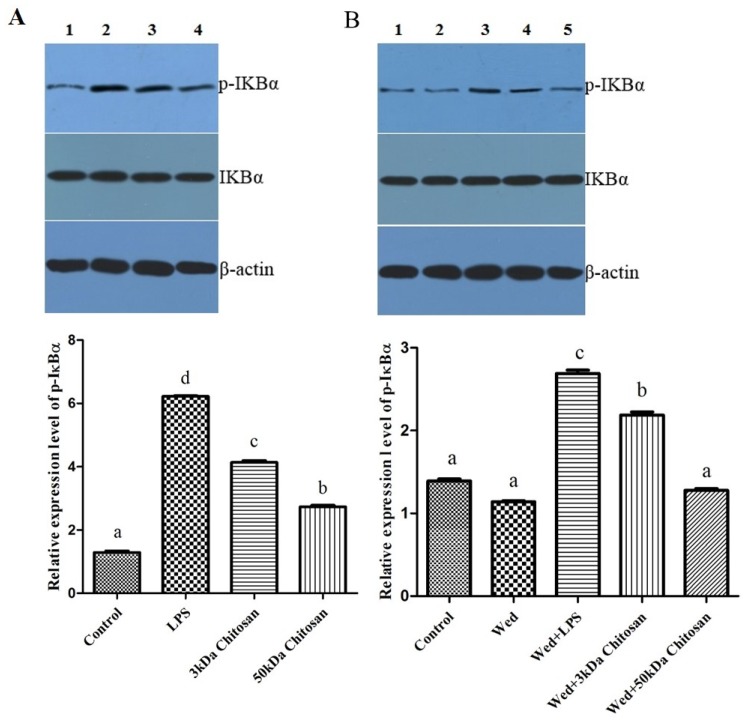
Effect of LMWCs on the phosphorylation of IKBα in RAW264.7 macrophage. (**A**) Each cell population (4 × 10^5^ cells/mL) was treated with LMWCs (3 kDa and 50 kDa) at the indicated concentrations of 40 μg/mL for 12 h, respectively (1: Control; 2: LPS; 3: 3 kDa chitosan; 4: 50 kDa chitosan); (**B**) Each cell population (4 × 10^5^ cells/mL) was treated with LMWCs (3 kDa and 50 kDa) at the indicated concentrations of 40 μg/mL and LPS (1 μg/mL) for 12 h after pre-incubation with 20 μmol/L of wedelolactone (Wed) for 12 h, respectively (1: Control; 2: Wedelolactone (Wed); 3: Wed + LPS; 4: Wed + 3 kDa chitosan; 5: Wed + 50 kDa chitosan). The figures shown are representative of three independent experiments. These represent mean values of three independent experiments. Values are presented as means ± SD (*n* = 3). Bars with different letters (a, b, c, d) are statistically different (*p* < 0.05).

As shown in Figure 4, LMWCs affected the phosphorylation of IKBα in the RAW264.7 cells. Compared with the untreated cells, LMWCs significantly increased the phosphorylation of IKBα when RAW264.7 cells were exposed to LMWCs at the indicated concentrations of 40 μg/mL for 12 h (Figure 4A); however, 3 kDa and 50 kDa chitosan did not increase significantly the level of IKBα. After cells were incubated with the IκB kinase (IKK) inhibitor wedelolactone (20 μmol/L) for 12 h, the results indicated that wedelolactone suppressed the phosphorylation of IKBα compared with untreated cells (Figure 4B), whereas 3 kDa chitosan induced significantly the phosphorylation of IKBα compared with 50 kDa chitosan (*p* < 0.05). Taken together, the results suggested that LMWCs significantly induced the phosphorylation of IKBα from RAW264.7 macrophage cells in a size-dependent manner.

### 2.5. Effects of LMWCs on the Protein Expression of p65 in RAW264.7 Macrophages

**Figure 5:**
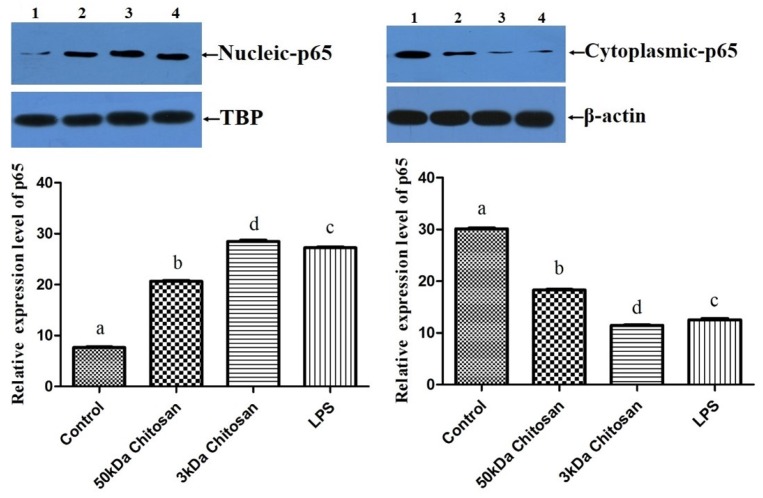
Effect of LMWCs on the nuclear translocation of p65 in the NF-κB pathway in RAW264.7 macrophages. Each cell population (1 × 10^6^ cells/mL) was treated with LMWCs (3 kDa and 50 kDa) at the indicated concentrations of 40 μg/mL for 24 h, respectively (1: Control; 2: 50 kDa chitosan; 3: 3 kDa chitosan; 4: LPS). The figures shown are representative of three independent experiments. These represent mean values of three independent experiments. Values are presented as means ± SD (*n* = 3). Bars with different letters (a, b, c, d) are statistically different (*p* < 0.05).

In order to examine whether the immunostimulative effects of LMWCs on RAW264.7 macrophages are associated with the translocation of p65 in the nuclear factor kB (NF-κB) pathway, the change in protein levels of NF-κB p65 in the cytoplasm and nucleus were investigated, as seen in Figure 5. As shown in Figure 5, treatment with LMWCs induced significant translocation of nucleic p65 protein and depletion of cytoplasmic p65, which is a subunit of NF-κB (*p* < 0.05). Moreover, the results indicated that treatment with 3 kDa chitosan significantly boosted the levels of nucleic p65 protein compared with 50 kDa chitosan (*p* < 0.05). These results suggested that immunostimulative effects of LMWCs might be associated with the nucleus translocation of p65 in the NF-κB pathway and that LMWCs activate macrophages via NF-κB signaling pathways in a molecular weight-dependent manner.

### 2.6. Effects of LMWCs on the Expression of AP-1 (C-Jun and C-Fos) in RAW264.7 Macrophages

**Figure 6:**
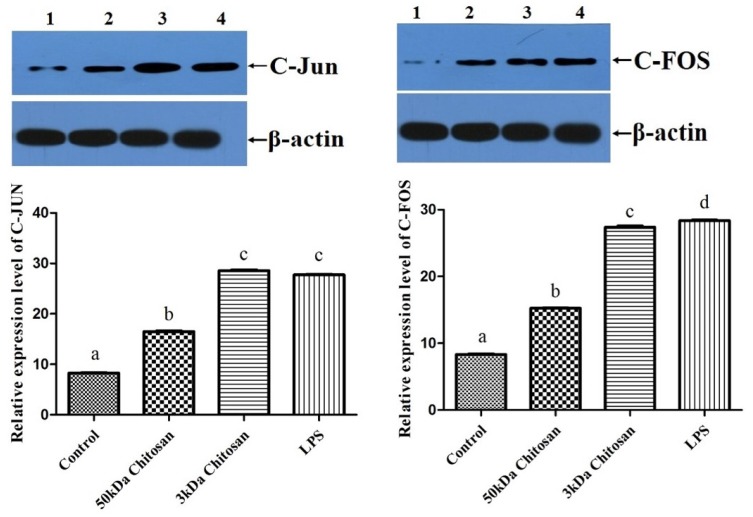
Effect of LMWCs on activator protein-1 (AP-1) in RAW264.7 macrophages. Each cell population (1 × 10^6^ cells/mL) was treated with LMWCs (3 kDa and 50 kDa) at the indicated concentrations of 40 μg/mL for 24 h, respectively (1: Control; 2: 50 kDa chitosan; 3: 3 kDa chitosan; 4: LPS). The figures shown are representative of three independent experiments. These represent mean values of three independent experiments. Values are presented as means ± SD (*n* = 3). Bars with different letters (a, b, c, d) are statistically different (*p* < 0.05).

In order to examine whether immunostimulative effects of LMWCs on RAW264.7 macrophages are associated with the expression of C-Jun and C-Fos proteins in the activator protein-1 (AP-1) pathway, change of protein levels of C-Jun and C-Fos in RAW264.7 macrophages were investigated in Figure 6. As shown in Figure 6, treatment with LMWCs induced significant up-regulation of the expression of C-Jun and C-Fos, which are two subunits of AP-1 (*p* < 0.05). Moreover, the results indicated that treatment with 3 kDa chitosan significantly increased the levels of C-Jun and C-Fos protein compared with 50 kDa chitosan (*p* < 0.05). These results suggested that the immunostimulative effects of 3 kDa chitosan might be associated with the expression activation of C-Jun and C-Fos proteins in RAW264.7 macrophage and LMWCs might activate macrophages via AP-1 signaling pathways in a molecular weight-dependent manner.

## 3. Discussion

**Figure 7:**
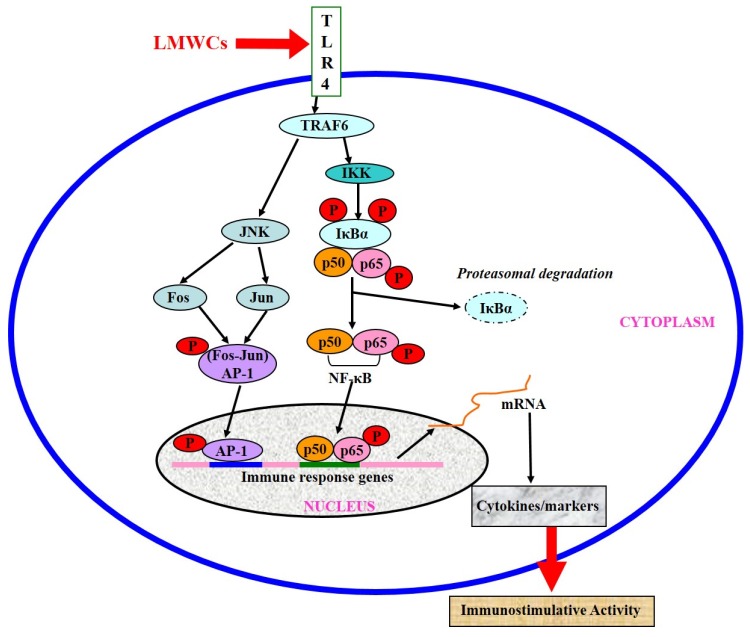
Schematic diagram of the targets of LMWCs.

Immunomodulatory activities of chitosan and its derivatives have been studied for their potential applications against allergy, infectious diseases or cancer [1,19]. Moreover, previous findings suggested that chitosan and its derivatives induce various inflammatory and pro-inflammatory cytokines upon incubating them with macrophages [20,21]. The immunostimulatory activity of LMWCs in non-induced RAW264.7 vary in a molecular weight-dependent manner because molecular weight might affect their structures and physicochemical properties. Previous studies reported that LMWCs have dual activities, both immunostimulatory activity in non-induced RAW264.7 [16] and anti-inflammatory activity in induced RAW264.7 cells [17]. LMWCs has previously been proved to provoke the immunomodulatory response through up-regulating mRNA expression of pro-inflammatory cytokines and activated RAW264.7 macrophages in a molecular weight-dependent manner [18]. However, its molecular mechanism responsible for regulating immune response is not fully understood. In the present study, the activation effect of LMWCs on the macrophages was investigated and its subsequent intracellular signaling pathways were explored using RAW264.7 macrophages as a cellular model. Our findings have demonstrated that LMWCs elicits an immunostimulative response in RAW264.7 macrophages through the simultaneous activation of the transcription factors NF-κB and AP-1 signaling pathways. Our hypothesized mode of action of LMWCs in this model of RAW264.7 macrophages is presented in Figure 7.

Macrophages actively participate in immune responses by releasing cytokines such as pro-inflammatory cytokines (TNF-α and IL-1) and inflammatory factors nitric oxide (NO) [22]. In the previous study, we found LMWCs significantly enhanced the pinocytic activity and induce the production of tumor necrosis factor α (TNF-α), interleukin 6 (IL-6), interferon-γ (IFN-γ), NO and inducible nitric oxide synthase (iNOS) in a molecular weight- and concentration-dependent manner [18]. Herein, LMWCs also significantly up-regulated the mRNA expression levels of prostaglandin-endoperoxide synthase 2 (COX-2), IL-10 and monocyte chemotactic protein-1 (MCP-1) in the same manner. The cytokines associated with polarized type Ι responses of activated M1 phenotypes include TNF-α, moreover, M2 cells typically produce IL-10 [23]. Differential production of chemokines integrates M1 and M2 macrophage in circuits of amplification to attract Th1 and Th2 or T regulatory (Treg) cells for inducing polarized T cell responses [24]. As discussed above, LMWCs significantly promoted the production of TNF-α and IL-10 from RAW264.7 macrophages. Taken together, the results suggested that LMWCs could simultaneously induce Th1- and Th2-type response in a molecular weight-dependent manner.

TNF receptor-associated factor (TRAF) proteins are also key components of activation of the immune system. During activation of macrophages, TRAF molecules autoubiquitinate through the E3 ubiquitin ligase in their RING domain [25]. Ubiquitination of TNF receptor-associated factor 6 (TRAF6) is a key regulatory event and often a target molecule for regulation by inhibitors of NF-κB [26]. TRAF6 may be activated through TLR4, which in turn activates the inhibitor of κB kinase, finally NF-κB will be activated [27]. NF-κB can be stimulated through Toll-like receptors (TLRs) to activate the IKK complex, leading to the translocation of heterodimers of the NF-κB subunits (p65 and p50) to the nucleus [28]. IKKβ is very important molecule for NF-κB activation in response to pro-inflammatory stimuli [29]. Most immune-stimulants activate the function of macrophages through binding specifically with the cell surface receptor proteins. TLR4 is known to be expressed on macrophages and other cells [30,31]. TLR4 signaling pathways may play important roles in immune cell activation. Previous studies reported that TLR4 on the cell membrane might mediate the biological effects of chitosan oligosaccharide on macrophages and the activation of murine spleen CD11c^+^ dendritic cells [32,33]. Moreover, Muzzarelli RA reported that the structure of chitosan is similar to the saccharide portion of lipid A in LPS, so it can similarly activate macrophages by binding to the surface TLR4 to initiate signal transduction [34]. To further insight into the molecular mechanism on immunomodulatory action of LMWCs, RT-PCR analysis showed that LMWCs directly significantly up-regulated the mRNA expression levels of IKKβ (Figure 2) and TRAF6 (Figure 3). Meanwhile, we also found that 3 kDa chitosan significantly promoted the mRNA expression levels of IKKβ compared with that at the same dose of 50 kDa chitosan, suggesting that LMWCs significantly induced the mRNA expression levels of IKKβ of macrophages dependent on its molecular weight. Our results suggested that the difference in NF-κB activated by LMWCs might be associated with the activation of TRAF6 and IKKβ in a molecular weight-dependent manner.

AP-1 is another important regulatory protein involved in cell growth, differentiation, transformation and apoptosis, moreover may also contribute to inflammatory and immune responses [35,36]. AP-1 is the target of mitogen-activated protein kinase (MAPK) signaling pathways through direct phosphorylation of AP-1 proteins [37,38]. In the immune response, AP-1 can regulate the production of cytokines such as TNF-α, IL-1, and IL-2 [39]. Previous studies suggested that JNK1 regulates the expression of pro-inflammatory cytokines and Nitric Oxide Synthase 2 (NOS2) during LPS and TNF-α activation, is a important molecule in macrophage biology [40,41]. Direct phosphorylation and transcriptional activation of AP-1 components by MAPKs lead to the stimulation of AP-1 activity [42]. Therefore, we further worked to determine whether LMWCs regulate the levels of TRAF6 and JNK1 in the macrophages. RT-PCR analysis showed that LMWCs directly significantly up-regulated the mRNA expression levels of TRAF6 and JNK1 (Figure 3), suggesting that LMWCs significantly induced the mRNA expression levels of TRAF6 of macrophages followed by promoting the expression levels of MAP Kinases JNK1 dependent on its molecule weight. Based on these findings, our results suggested that LMWCs might modulate the transcriptional activities of AP-1 by regulating levels of TRAF6 and JNK1 in macrophages.

Several lines of evidence indicate that LMWC plays an important role in the regulation of inflammatory responses through NF-κB and AP-1 signaling pathway [43,44,45]. Activation of NF-κB in response to stimuli involves activation of IκBα kinase (IKK), phosphorylation and degradation of IκBα, followed by release of activated NF-κB. The active dimer translocate to the nucleus where it binds to its target DNA sequence and induces the expression of its downstream genes [46,47]. Some of the best characterized substrates of the JNKs are the components of AP-1, a dimeric transcription factor formed by the association of Fos protein (c-Fos, FosB, Fra-1 and Fra-2) with Jun proteins (c-Jun, JunB and JunC). Both the chemical structure and the molecular size of LMWC might affect the NF-κB and AP-1 activation efficacy, followed by the activation of macrophages. Bahar et al. (2012) reported that chitooligosaccharide elicits an acute inflammatory cytokine response in Caco-2 cells through the simultaneous activation of the AP-1 transcription factor pathway [45]. Li et al. (2012) found that all five chitosan oligosaccharides (chitobiose, chitotriose, chitotetraose, chitopentaose, chitohexaose) increased NF-κB-dependent luciferase gene expression and NF-κB downstream genes transcription, and the most significant were chitotetraose and chitohexaose. In addition, they activated the p65 subunit of NF-κB translocating from cytoplasm to nucleus, which suggested that they were the most potent activators of the NF-κB signaling pathway [21,44]. In this regard, this study was carried out to investigate effects of LMWCs on the NF-κB and AP-1 signaling pathways and the expression of its downstream genes. Our data presented here demonstrated that LMWCs (3 kDa and 50 kDa chitosan) were the most potent activators of NF-κB and AP-1 signaling pathway and initiators of their downstream genes transcription. In addition, both of them also activated the p65 subunit of NF-κB p65 and AP-1 translocating from cytoplasm to nucleus in a molecular weight-dependent manner (Figure 5 and Figure 6). Moreover, we assessed whether activation of translocation of the NF-κB p65 subunit is attributed to promoting the phosphorylation of IκBα by LMWCs. The results indicated that LMWCs could increase the phosphorylation of IκBα followed by the activation of degradation of IκBα in the cytoplasm in a molecular weight-dependent manner (Figure 4). Based on these findings, the results suggested that LMWCs activate the p65 subunit of NF-κB p65 translocating from cytoplasm to nucleus by promoting the phosphorylation of IκBα in a molecular weight-dependent manner.

In summary, the results presented in this study suggested that NF-κB and AP-1 signaling pathways were involved in the macrophage activation by two different molecular weights of LMWC. It is assumed that activation of TRAF6, JNK1, IKKβ and IκBα and subsequent activation of transcription factors (NF-κB and AP-1) were the main mechanism involved in the macrophage activation by LMWCs in a molecular weight-dependent manner. Taken together, our findings suggest that molecular weight affects the immuostimulative activity of LMWC via NF-κB and AP-1 pathways and that 3 kDa LMWCs show great potential as a novel agent for the treatment of immune suppression diseases and as an adjuvant in future vaccines.

## 4. Experimental Section

### 4.1. Chemicals and Reagents

Dulbecco’s modified Eagle’s medium (DMEM), penicillin/streptomycin, and the other materials required for culture of cells were purchased from Gibco BRL, Life Technologies (Grand Island, NY, USA). Vitamin C, dimethylsulfox-ide (DMSO), 3-(4,5-dimethylthiazol-2-yl) 2,5-diphenyltetrazolium bromide (MTT), wedelolactone and bovine serum albumin (BSA) were obtained from Sigma (St. Louis, MO, USA). Trizol was from Invitrogen (Carlsbad, CA, USA), revert Aid™ M-MuLV reverse transcriptase was from Fermentas (Amherst, NY, USA), diethylpyrocarbonate (DEPC) and ribonuclease inhibitor were from Biobasic, Canada, oligo (dT)_18_ were from Sangon, China. Power SYBR^®^ Master Mix was from Invitrogen, Carlsbad, CA, USA. Super Signal^®^ West Dura Extended Duration Substrate, NE-PER^®^ Nuclear and cytoplasmic extraction reagents and BCA™ protein assay kit were purchased from Pierce, Rockford, IL, USA. Polyclonal antibodies (Abs) against NF-κB p65 and monoclonal antibody against AP-1 (C-fos and C-jun) and β-actin were from Santa Cruz Biotechnology, Dallas, Texas, USA. Monoclonal antibodies against IκB-α, p-IκB-α and TATA binding protein TBP were from abcam, Cambridge, CB4 0FL, UK. X-ray films were from Kodak, Rochester, NY, USA. All other chemicals were of analytical grade or of the highest grade available commercially.

The low molecular weight chitosans were sterilized by passing it through a 0.22-μm Millipore filter to remove any contaminant and then analyzed for endotoxin level by a gel-clot Limulus amebocyte lysate assay (Zhejiang A and C Biological, Zhejiang, China). The endotoxin level in the stock solution was less than 0.5 EU/mL.

### 4.2. Cell Culture and Treatment

Mouse macrophages RAW 264.7 cell line was obtained from the Shanghai Institute of Cell Biology (Shanghai, China) and maintained in DMEM, supplemented with heat-inactivated 10% fetal bovine serum, 100 U/mL penicillin, 100 U/mL streptomycin in a humidified atmosphere of 5% CO_2_ at 37 °C. When the cells reached sub-confluence, they were treated for 12 or 24 h with culture medium containing different concentrations of LMWCs, two MW, 3 kDa and 50 kDa (2.5, 10, and 40 μg/mL), and LPS (1 μg/mL) that were tested in the experiments.

### 4.3. Real-Time Fluorescent Quantitative Reverse Transcription-Polymerase Chain Reaction (RT-PCR) Analysis

After incubation with or without LMWCs, RAW264.7 cells were lysed in 1 mL of Trizol reagent (Invitrogen™, Carlsbad, CA, USA) and the total RNA was isolated according to the manufacture’s protocol. The concentration of total RNA was quantified by determining the optical density at 260 nm. The total RNA was used and reverse transcription (RT) was performed using 1st-Strand cDNA Synthesis Kit (Invitrogen™, Carlsbad, CA, USA). Briefly, nuclease-free water was added giving a final volume of 5 μL after mixing 2 μg of RNA with 0.5 μg oligo (dT)_18_ primer in a DEPC-treated tube. This mixture was incubated at 65 °C for 5 min and chilled on ice for 2 min. Then, a solution containing 3 μL of RT buffer Mix, 0.65 μL of RT Enzyme Mix and 1.35 uL Primer Mix, giving a final volume of 10 μL, and the tubes were incubated for 10 min at 30 °C. The tubes then were incubated for 30 min at 42 °C. Finally, the reaction was stopped by heating at 70 °C for 15 min. The samples were stored at −20 °C until further use.

As shown in Table 1, the primers were used to amplify cDNA fragments (144-bp COX2 fragment, 144-bp IL-10 fragment, 101-bp MCP-1 fragment, 73-bp TRAF6 fragment, 109-bp JNK1 fragment, 104-bp IKKβ fragment and 94-bp 18S fragment). Amplification was carried out as previously [18] in total volume of 25 μL containing 1 μL (5 μM) of each target and 18S specific primers, 1 μL of cDNA template, 12.5 μL of Power SYBR^®^ Master Mix (2×) (4 μL of 10× PCR buffer, 4 μL of MgCl_2_ (25 mM), 4 μL of dNTPs (2.5 mM) and 0.5 μL of Taq DNA polymerase) (Invitrogen, USA), and 10.5 μL of DEPC-treated water was added. Reaction conditions were the standard conditions for the iQTM5 PCR (Bio-Rad, Hercules, CA, USA) (10 s denaturation at 95 °C, 25 s annealing at 63 °C or 64 °C (COX2, IL-10, MCP-1, TRAF6, JNK1, IKKβ, 18S) with 40 PCR cycles. Ct values were obtained automatically using software (Bio-Rad, USA). The comparative Ct method (2^−ΔCt^ method) [48] was used to analyze the expression levels of genes, and 18S rRNA was used as the house-keeping gene.

### 4.4. Western Blot Analysis

After treated with the various concentrations of LMWCs, RAW264.7 cells were washed three times with cold PBS and lysed with NE-PER^TM^ nuclear and cytoplasmic extraction reagents (Pierce, Rockford, IL, USA). The protein contents were measured with the BCA protein assay kit using bovine serum as a standard. The denatured proteins were separated on 10%–12% sodium dodecyl sulfate polyacrylamide gel electrophoresis (SDS-PAGE), and transferred to PVDF membrane. After blocking the membrane with 5% skim milk in tween-20 containing Tris buffered saline (T-TBS) (20 mM Tris-HCl (pH 7.6), 150 mM NaCl, 0.1% Tween-20) for 1 h at 37 °C, the blots were incubated with mouse monoclonal antibody p-IκBα (1:1000), IκBα (1:2000), TBP (1:800), β-actin (1:1000), C-Fos (1:300) and rabbit polyclonal NF-κB p65 (1:500), C-Jun (1:400) in T-TBS containing 3% skim milk overnight at 4 °C. Subsequently, the membranes were washed with TTBS and incubated with an appropriate secondary antibody (horseradish peroxidase-conjugated goat anti-mouse or anti-rabbit IgG) for 1 h. After washing the membrane with T-TBS five times for 5 min, the signal was visualized with ECL Detection Kit (SuperSignal^®^ West Dura Extended Duration Substrate) and exposed the membranes to X-ray films. The bands were visualized and photographed using JS-680B Gel Documentation and Analysis System. The relative expression levels of the proteins were expressed as 100 (or 10) × the gray value of the target protein band over the gray value of β-actin or TBP in the same sample. Each sample had 3 replicates.

### 4.5. Statistical Analysis

Data were expressed as mean ± standard deviation (S.D.) and examined for their statistical significance of difference with ANOVA and a Tukey post hoc test by using SPSS 16.0. *p*-values of less than 0.05 were considered statistically significant.

## Figures and Tables

**Table 1:** Real-Time PCR Primers and Conditions.

Gene	Genbank Accession	Primer Sequence	Product Size (bp)	Annealing (°C)
COX2	NM_011198.3	TCTGGCTTCGGGAGCACAAC	144	64
		GGTGTTGCACGTAGTCTTCGATCA		
IL-10	NM_010548.2	CAGTCGGCCAGAGCCACAT	144	64
		CTTGGCAACCCAAGTAACCCTT		
MCP-1	NM_011333.3	CTGCATCTGCCCTAAGGTCTTCA	101	64
		AGTGCTTGAGGTGGTTGTGGAA		
TRAF6	D84655.1	GGAGGACAAGGTTGCCGAAAT	73	63
		CCCAAACTTGCCAATCTTCCAA		
JNK1	NM_001310454.1	GCTCTCCAGCACCCATACATCA	109	63
		CCTCTATTGTGTGCTCCCTCTCAT		
IKKβ	AF088910.1	CAGAAGTACACCGTGACCGTTGA	104	63
		CACTGCACAGGCTGCCAGTTA		
18S	NR_003278	CGGACACGGACAGGATTGACA	94	64
		CCAGACAAATCGCTCCACCAACTA		

## References

[1] Zaharoff D.A., Rogers C.J., Hance K.W., Schlom J., Greiner J.W. (2007). Chitosan solution enhances both humoral and cell-mediated immune responses to subcutaneous vaccination. Vaccine.

[2] Read R.C., Naylor S.C., Potter C.W., Bond J., Jabbal-Gill I., Fisher A., Illum L., Jennings R. (2005). Effective nasal influenza vaccine delivery using chitosan. Vaccine.

[3] Mills K.H., Cosgrove C., McNeela E.A., Sexton A., Giemza R., Jabbal-Gill I., Church A., Lin W., Illum L., Podda A. (2003). Protective levels of diphtheria-neutralizing antibody induced in healthy volunteers by unilateral priming-boosting intranasal immunization associated with restricted ipsilateral mucosal secretory immunoglobulin a. Infect. Immun..

[4] McNeela E.A., Jabbal-Gill I., Illum L., Pizza M., Rappuoli R., Podda A., Lewis D.J., Mills K.H. (2004). Intranasal immunization with genetically detoxified diphtheria toxin induces T cell responses in humans: Enhancement of Th2 responses and toxin-neutralizing antibodies by formulation with chitosan. Vaccine.

[5] Wen Z.-S., Xu Y.-L., Zou X.-T., Xu Z.-R. (2011). Chitosan Nanoparticles Act as an Adjuvant to Promote both Th1 and Th2 Immune Responses Induced by Ovalbumin in Mice. Mar. Drugs.

[6] Eun J.J., Dal K.Y., Shin H.L., Hong K.N., Jong G.H., Prinyawiwatkul W. (2010). Antibacterial activity of chitosans with different degrees of deacetylation and viscosities. Int. J. Food Sci. Technol..

[7] Artan M., Karadeniz F., Karagozlu M.Z., Kim M.M., Kim S.K. (2010). Anti-HIV-1 activity of low molecular weight sulfated chitooligosaccharides. Carbohydr. Res..

[8] Fernandes J.C., Spindola H., de Sousa V., Santos-Silva A., Pintado M.E., Malcata F.X., Carvalho J.E. (2010). Anti-inflammatory activity of chitooligosaccharides in vivo. Mar. Drugs.

[9] Ngo D.N., Lee S.H., Kim M., Kim S.K. (2009). Production of chitin-oligosaccharides with different molecular weights and their antioxidant effect in RAW264.7 cells. J. Funct. Foods.

[10] Ju C.X., Yue W., Yang Z.H., Zhang Q., Yang X., Liu Z., Zhang F. (2010). Antidiabetic effect and mechanism of chitooligosaccharides. Biol. Pharm. Bull..

[11] Gong Y., Gong L., Gu X., Ding F. (2009). Chitooligosaccharides promote peripheral nerve regeneration in a rabbit common peronial nerve crush injury model. Microsurgery.

[12] Quan H., Zhu F., Han X., Xu Z., Zhao Y., Miao Z. (2009). Mechanism of anti-angiogenic activities of chitooligosaccharides may be through inhibiting heparanase activity. Med. Hypothesis.

[13] Lee C.G., Da Silva C.A., Lee J.Y., Hartl D., Elias J.A. (2008). Chitin regulation of immune responses: An old molecule with new roles. Curr. Opin. Immunol..

[14] Li X., Min M., Du N., Gu Y., Hode T., Naylor M., Chen D., Nordquist R.E., Chen W.R. (2013). Chitin, Chitosan, and Glycated Chitosan Regulate Immune Responses: The Novel Adjuvants for Cancer Vaccine. Clin. Dev. Immunol..

[15] Suzuki K., Mikami T., Okawa Y., Tokoro A., Suzuki S., Suzuki M. (1986). Antitumor effect of hexa-*N*-acetylchitohexaose and chitohexaose. Carbohydr. Res..

[16] Okamoto Y., Inoue A., Miyatake K., Ogihara K., Shigemasa Y., Minami S. (2003). Effects of chitin/chitosan and their oligomers/monomers on migrations of macrophages. Macromol. Biosci..

[17] Yang E.J., Kim J.G., Kim J.Y., Kim S.C., Lee N.H., Hyun C. (2009). Anti-inflammatory effect of chitosan oligosaccharides in RAW264.7 cells. Cent. Eur. J. Biol..

[18] Wu N., Wen Z., Xiang X., Huang Y., Gao Y., Qu Y. (2015). Immunostimulative activity of low molecular weight chitosans in RAW264.7 macrophages. Mar. Drugs.

[19] Chen Y.L., Wang C.Y., Yang F.Y., Wang B.S., Chen J.Y., Lin L.T., Leu J.D., Chiu S.J., Chen F.D., Lee Y.J. (2014). Synergistic effects of glycated chitosan with high-intensity focused ultrasound on suppression of metastases in a syngeneic breast tumor model. Cell Death Dis..

[20] Da Silva C.A., Chalouni C., Williams A., Hartl D., Lee C.G., Elias J.A. (2009). Chitin is a size-dependent regulator of macrophage TNF and IL-10 production. J. Immunol..

[21] Bueter C.L., Lee C.K., Rathinam V.A., Healy G.J., Taron C.H., Specht C.A., Levitz S.M. (2011). Chitosan but not chitin activates the inflammasome by a mechanism dependent upon phagocytosis. J. Biol. Chem..

[22] Farias-Eisner R., Sherman M.P., Aeberhard E., Chaudhuri G. (1994). Nitric oxide is an important mediator for tumoricidal activity in vivo. Proc. Natl. Acad. Sci. USA.

[23] Rauh M.J., Ho V., Pereira C., Sham A., Sly L.M., Lam V., Huxham L., Minchinton A.I., Mui A., Krystal G. (2005). SHIP represses the generation of alternatively activated macrophages. Immunity.

[24] Mantovani A., Sozzani S., Locati M., Allavena P., Sica A. (2002). Macrophage polarization: Tumor-associated macrophages as a paradigm for polarized M2 mononuclear phagocytes. Trends Immunol..

[25] Bishop G.A. (2004). The multifaceted roles of TRAFs in the regulation of B-cell function. Nat. Rev. Immunol..

[26] Schneider M., Zimmermann A.G., Roberts R.A., Zhang L., Swanson K.V., Wen H., Davis B.K., Allen I.C., Holl E.K., Ye Z. (2012). The innate immune sensor NLRC3 attenuates Toll-like receptor signaling via modification of the signaling adaptor TRAF6 and transcription factor NF-κB. Nat. Immunol..

[27] Yin Q., Lin S.C., Lamothe B., Lu M., Lo Y.C., Hura G., Zheng L., Rich R.L., Campos A.D., Myszka D.G. (2009). E2 interaction and dimerization in the crystal structure of TRAF6. Nat. Struct. Mol. Biol..

[28] Hasegawa M., Fujimoto Y., Lucas P.C., Nakano H., Fukase K., Núñez G., Inohara N. (2008). A critical role of RICK/RIP2 polyubiquitination in Nod-induced NF-κB activation. EMBO J..

[29] Karin M., Delhase M. (2000). The IκB kinase (IKK) and NF-κB: Key elements of proinflammatory signaling. Semin. Immunol..

[30] Beutler B. (2000). Tlr4: Central component of the sole mammalian LPS sensor. Curr. Opin. Immunol..

[31] Janeway C.A., Medzhitov R. (2002). Innate immune recognition. Annu. Rev. Immunol..

[32] Zhang P., Liu W., Peng Y., Han B., Yang Y. (2014). Toll like receptor 4 (TLR4) mediates the stimulating activities of chitosan oligosaccharide on macrophages. Int. Immunopharmacol..

[33] Dang Y., Li S., Wang W., Wang S., Zou M., Guo Y., Fan J., Du Y., Zhang J. (2011). The effects of chitosan oligosaccharide on the activation of murine spleen CD11c+ dendritic cells via Toll-like receptor 4. Carbohydr. Polym..

[34] Muzzarelli R.A. (1997). Human enzymatic activities related to the therapeutic administration of chitin derivatives. Cell. Mol. Life Sci..

[35] Angel P., Karin M. (1991). The role of Jun, Fos and the AP-1 complex in cell-proliferation and transformation. Biochim. Biophys. Acta.

[36] Shaulian E., Karin M. (2001). AP-1 in cell proliferation and survival. Oncogene.

[37] Whitmarsh A.J., Davis R.J. (1996). Transcription factor AP-1 regulation by mitogen-activated protein kinase signal transduction pathways. J. Mol. Med..

[38] Pearson G., Robinson F., Beers Gibson T., Xu B.E., Karandikar M., Berman K., Cobb M.H. (2001). Mitogen-activated protein (MAP) kinase pathways: Regulation and physiological functions. Endocr. Rev..

[39] Giri R.S., Thaker H.M., Giordano T., Williams J., Rogers D., Sudersanam V., Vasu K.K. (2009). Design, synthesis and characterization of novel 2-(2,4-disubstituted-thiazole-5-yl)-3-aryl-3Hquinazoline-4-one derivatives as inhibitors of NF-κB and AP-1 mediated transcription activation and as potential anti-inflammatory agents. Eur. J. Med. Chem..

[40] Sánchez-Tilló E., Comalada M., Xaus J., Farrera C., Valledor A.F., Caelles C., Lloberas J., Celada A. (2007). JNK1 Is required for the induction of Mkp1 expression in macrophages during proliferation and lipopolysaccharide-dependent activation. J. Biol. Chem..

[41] Guma M., Ronacher L.M., Firestein G.S., Karin M., Corr M. (2011). JNK1 deficiency limits macrophage mediated antigen-induced arthritis. Arthritis Rheumatol..

[42] Wu H., Arron J.R. (2003). TRAF6, a molecular bridge spanning adaptive immunity, innate immunity and osteoimmunology. Bioessays.

[43] Wu G.J., Tsai G.J. (2007). Chitooligosaccharides in Combination with Interferon-γ Increase Nitric Oxide Production via Nuclear Factor-κB Activation in Murine RAW264.7 Macrophages. Food Chem. Toxicol..

[44] Li X., Zhou C., Chen X., Wang J., Tian J. (2012). Effects of Five Chitosan Oligosaccharides on Nuclear Factor-kappa B Signaling Pathway. J. Wuhan Univ. Technol.—Mater. Sci. Ed..

[45] Bahar B., O’Doherty J.V., Maher S., McMorrow J., Sweeney T. (2012). Chitooligosaccharide elicits acute inflammatory cytokine response through AP-1 pathway in human intestinal epithelial-like (Caco-2) cells. Mol. Immunol..

[46] Moynagh P.N. (2005). The NF-kappaB Pathway. J. Cell Sci..

[47] Hayden M.S., Ghosh S. (2008). Shared Principles in NF-κB Signaling. Cell.

[48] Livak K.J., Schmittgen T.D. (2001). Analysis of Relative Gene Expression Data Using Real-Time Quantitative PCR and the 2^−ΔCt^ Method. Methods.

